# Patient-reported outcome measures more than fifteen years after treatment of sagittal or metopic craniosynostosis: a prospective cohort study

**DOI:** 10.1007/s00381-023-06202-w

**Published:** 2023-11-02

**Authors:** Peter A. Woerdeman, Vita M. Klieverik, Alexander Cheong, Ash Singhal, Douglas Cochrane, Paul Steinbok

**Affiliations:** 1 Department of Neurology and Neurosurgery, University Medical Center Utrecht, Heidelberglaan 100, 3584 CX Utrecht, The Netherlands; 2 Center for Disease Control, Faculty of Medicine, University of British Columbia, Vancouver, BC Canada; 3 Division of Pediatric Neurosurgery, Division of Neurosurgery, British Columbia Children’s Hospital, University of British Columbia, Vancouver, BC Canada

**Keywords:** Craniosynostosis, Long-term, Cosmetic satisfaction, Patient-reported outcome measures

## Abstract

**Purpose:**

To evaluate the long-term anthropometric measurements, cosmetic satisfaction, and other patient-reported outcome measures (PROMs) of patients who underwent surgical treatment or observation only of sagittal or metopic single-suture craniosynostosis (SSC).

**Methods:**

A prospective study was designed for all patients diagnosed with non-syndromic sagittal and metopic craniosynostosis at the British Columbia Children’s Hospital, Vancouver, Canada, in the period July 1986 to July 2006. After a minimum of 15 years post-diagnosis, all eligible patients were invited to fill out the Craniofacial Surgery Outcomes Questionnaire (CSO-Q) and to attend a scheduled follow-up appointment for the collection of anthropometric measurements. A descriptive analysis of the cosmetic results was performed. Statistical analyses compared the differences in anthropometric measurements between treated and non-treated patients.

**Results:**

Of the 253 eligible patients, 52 participants were willing to share patient data for use in the study. Of those 52 former patients, 36 (69.2%) filled out and returned the CSO-Q and 23 (44.2%) attended the follow-up appointment. The mean follow-up period between surgical treatment and the CSO-Q was 20.2 ± 2.5 years and between surgical treatment and the follow-up appointment was 20.9 ± 2.7 years. In patients with sagittal SSC, the mean cephalic index (CI) was significantly larger in treated than in non-treated patients (74.6 versus 69.1, p = 0.04), while the mean pupillary distance and forehead to back index were significantly smaller (pupillary distance 6.0 cm versus 6.7 cm [p = 0.04] and forehead to back index 19.6 cm versus 21.1 cm [p = 0.03]). Focusing more on the patient reported outcome measures, overall cosmetic satisfaction was found to be high (80.6%) and no differences were found between sagittal and metopic synostosis patients, nor between treated or non-treated craniosynostosis patients. Overall outcomes regarding self-esteem (RSES) and fear of negative evaluation (FNE) were comparable with population based outcomes.

**Conclusion:**

This is the first prospective study of sagittal and metopic craniosynostosis patients regarding long-term anthropometric outcome and patient reported outcome measures, including patients who were treated surgically and those who received observation only. Although study participation two decades after initial diagnosis was difficult to obtain, our data provide a platform from which one can develop an inclusive and uniform approach to assess patients’ subjective cosmetic satisfaction using the CSO-Questionnaire and might be useful in preoperative counseling and psychosocial care for patients and their families.

**Supplementary Information:**

The online version contains supplementary material available at 10.1007/s00381-023-06202-w.

## Introduction

Craniosynostosis is a congenital condition that involves premature and pathological fusion of one or multiple cranial sutures [1]. The incidence of craniosynostosis is around 1 in 2500 live births [2]. Craniosynostosis mainly presents as an isolated defect of one cranial suture without other associated abnormalities, known as single-suture craniosynostosis (SSC) [1, 2]. SSC is often surgically treated to correct and prevent severe cosmetic deformity and to prevent possible impairments in brain and cognitive development [3]. After surgical treatment, patients are usually followed up until early childhood. Generally, this follow-up does not extend past school age because late sequelae of neuropsychological and cognitive impairments are usually not expected after surgical treatment [4, 5]. Nevertheless, patients with SSC may suffer from psychosocial difficulties [4]. A retained cosmetic deformity might unfavorably affects patients’ level of self-esteem, sense of belonging, social behavior and experience, and overall health-related quality of life [4]. Therefore, evaluation of anthropometric measurements and patients’ subjective assessment of their skull and facial appearance is important [6]. Furthermore, patient-reported outcome measures (PROMs) such as self-esteem and fear of negative evaluation (FNE) are other valuable outcome measures to evaluate, both in early childhood as well as later on in life. Data on these PROMs are useful for patients’ families and physicians to incorporate in preoperative discussions on the most optimal management of SSC and postoperative psychosocial care [5]. However, uniform long-term follow-up data on anthropometric measurements, cosmetic satisfaction, and other PROMs of patients who underwent surgical treatment of SSC are scarce [6]. Therefore, the objective of this study was to evaluate the long-term anthropometric measurements, cosmetic satisfaction, and other PROMs of these former patients and to provide an inclusive and uniform tool for assessing their long-term postoperative outcomes.

## Methods

### Study design and study population

Utilizing the prospectively maintained Neurosurgical Database at BC Children’s Hospital (BCCH) (Vancouver, British Columbia), we identified all patients diagnosed with SSC from July 1986 to July 2006. BCCH serves as the referral center for Pediatric Neurosurgery for a population of approximately 3.5 million-5 million over the span of the study, with approximately 40,000–50,000 live births annually. Patients were eligible for inclusion if they were diagnosed with sagittal or metopic craniosynostosis. Patients were excluded if they were also diagnosed with coronal, syndromic or multiple-suture craniosynostosis. Patients who were operated upon underwent a variety of surgical procedures for SCS, all of which had in common a vertex craniotomy of variable width with or without parietal widening, frontal and or occipital remodeling. A frontal orbital advancement with frontal bone recontouring or transposition was performed for those with metopic synostosis. Post operative helmeting was not used to affect shape change. This study was reviewed and approved by the institution’s Medical Research Ethics Committee (MREC) and all patients provided written informed consent.

### Data collection

After a minimum of 15 years follow-up, both treated and non-treated patients diagnosed with sagittal or metopic craniosynostosis were invited to participate in the study which involved completing the Craniofacial Surgery Outcomes Questionnaire (CSO-Q see appendix) and to attend a scheduled follow-up appointment at the outpatient clinic. The CSO-Q was used to collect data regarding cosmetic satisfaction and other PROMs. All responses were registered anonymously. During the follow-up appointment, data on anthropometric measurements were collected. The anthropometric measurements that were studied included the cephalic index (CI), pupillary distance, forehead to back index, head circumference, ear to ear distance, ear to ear index, and glabella-opisthocranion distance.

#### Composition of the craniofacial surgery outcomes questionnaire

The introductory part of the CSO-Q contains questions regarding demographic information, including patients’ general health and social situation. Part A of the CSO-Q includes an assessment of patients’ satisfaction with the aesthetic appearance of their face and skull. This assessment contains 6 questions which can be answered on a 5-point Likert scale (very satisfied, 4 points; satisfied, 3 points; neutral, 2 points; not satisfied, 1 point; and not satisfied at all, 0 points). Part B of the CSO-Q consists of the Rosenberg Self-Esteem Scale (RSES), the most widely used and validated scale to measure individuals’ level of self-esteem [7]. The RSES contains 10 statements for which patients need to decide whether they agree or disagree using a 4-point Likert scale (strongly agree, 3 points; agree, 2 points; disagree, 1 point; strongly disagree, 0 points). Statements 3, 5, 8, 9, and 10 are reverse scored. Part C of the CSO-Q includes an assessment of patients’ feelings of noticeability of their facial and skull appearance to others. This assessment contains 3 questions which can be answered on a 3-point Likert scale (often, 2 points; sometimes, 1 point; never, 0 points). Part D of the CSO-Q consists of the FNE scale, a standardized and validated tool to measure anxiety associated with perceived negative evaluation [8]. The FNE scale contains 30 true-false statements of which 17 are straightforwardly-worded (directly scored) and 13 are reverse-worded (reverse scored). The full CSO-Q is provided in the Supplementary Material.

### Statistical analysis

A descriptive analysis of the anthropometric measurements and the results of the CSO-Q was performed. Normally distributed continuous data were presented as means ± standard deviations (SD). Skewed distributed continuous data were expressed as medians with corresponding interquartile ranges (IQR). Categorical data were shown as numbers with corresponding percentages. Likert-scale data were analyzed and visualized using stacked bar charts. Results of part A of the CSO-Q were dichotomized into cosmetic satisfaction (very satisfied or satisfied with at least 4 out of 6 questions) and cosmetic dissatisfaction. Based on the results of the RSES, patients were categorized into high self-esteem (score of 26 to 30 points), normal self-esteem (score of 15 to 25 points), and low self-esteem (score of < 15 points). Based on the results of the FNE scale, patients were grouped into low fear (score of ≤ 12 points), average fear (score of 13 to 20 points), and high fear (score of 21 to 30 points). To test for differences in anthropometric measurements of treated versus non-treated patients diagnosed with sagittal or metopic craniosynostosis, independent samples T-tests for differences in means and Mann-Whitney U tests for differences in medians were performed. Potential correlations between outcomes of part A of the CSO-Q and the anthropometric measurements were evaluated using the Spearman correlation. Statistical significance was defined as p-value < 0.05. All statistical analyses were performed using R statistical software, version 4.0.2. (R Foundation for Statistical Computing, Vienna, Austria).

## Results

### Patient characteristics

Over the period 1986–2006, we retrieved 253 consecutive patients from the BCCH patient records who were diagnosed with sagittal (n = 208) or metopic (n = 45) single suture craniosynostosis (Fig. 1A).

Of these 253 patients, 52 patients were willing to share their data for study purposes and comprise the study population(Fig. 1B). The patient characteristics are shown in Table 1. Of the 52 patients in the study population, 39 (75.0%) were male and the median age at diagnosis was 3.0 months (IQR 1.6 to 9.0 months). Forty-one patients (78.8%) received surgical treatment for their single suture craniosynostosis and 11 patients (21.2%) received observation only. The median age at surgical treatment was 5.0 months (IQR 3.5 to 10.3 months).

**Table 1:** Baseline characteristics of 52 patients at time of SSC diagnosis (all data given as number of patients (%) unless otherwise indicated)

Characteristic	Value
Males	39 (75.0)
Median age at diagnosis (IQR, months)	3.0 (1.6–9.0)
Diagnosis	
Sagittal SSC	39 (75.0)
Metopic SSC	13 (25.0)
Median age at surgical treatment (IQR, months)	5.0 (3.5–10.3)
Surgical treatment	
Yes	41 (78.8)
No	11 (21.2)
Surgical treatment for sagittal SSC	34 (65.4)
Strip craniectomy	1 (1.9)
With biparietal wedges	13 (25.0)
With biparietal wedges and occipital craniectomy	4 (7.7)
With biparietal and bifrontal wedges and occipital craniectomy	1 (1.9)
With biparietal wedges and occipital, bicoronal and bisphenoidal craniectomy	1 (1.9)
With biparietal osteotomies and bifrontal remodeling	3 (5.8)
With biparietal osteotomies and occipital craniectomy	3 (5.8)
With biparietal widening cranioplasty and occipital craniectomy	1 (1.9)
With bifrontal wedges and lambdoidal and squamosal suturectomies	1 (1.9)
With bifrontal remodeling	1 (1.9)
With bifrontal remodeling and occipital craniectomy	1 (1.9)
Cranial vault reconstruction	1 (1.9)
Bilateral parietal craniectomy	1 (1.9)
Vertex craniectomy	2 (3.8)
Surgical treatment for metopic SSC	7 (13.5)
Bifrontal craniotomy and orbital advancement	7 (13.5)

Missing values were present for age at diagnosis (3.8%), age at surgical treatment (9.6%). SSC = single-suture craniosynostosis, IQR = interquartile range

**Table 2A:** Anthropometric measurements at follow up of treated versus non-treated patients diagnosed with sagittal SSC (N = 39)

Outcomes	Treated patients (n = 13)*	Non-treated patients (n = 4)*	p-value	
Head circumference	57.3	59.8	0.06^**†**^	
CI	74.6	69.1	0.04^**†**^	
Ear to ear distance	30.2	30.0	0.81^**†**^	
Ear to ear index	14.6	14.5	0.81^**†**^	
Pupillary distance	6.0	6.7	0.04^**†**^	
Glabella-opisthocranion distance	35.2	36.5	0.50^‡^	
Forehead to back index	19.6	21.1	0.03^**†**^	

* Results of patients with available data for the anthropometric measurements. Total number of treated patients was 34 and total number of non-treated patients was 5. ^**†**^ p-value for independent samples T-test for difference in means. ^‡^ p-value for Mann-Whitney U test for difference in medians. Head circumference, ear to ear distance, ear to ear index, pupillary distance, glabella-opisthocranion distance and forehead to back index are measured in centimeters. CI = cephalic index

**Table 2B:** Anthropometric measurements at follow up of treated versus non-treated patients diagnosed with metopic SSC (N = 13)

Outcomes	Treated patients (n = 3)*	Non-treated patients (n = 3)*	p-value	
Head circumference	56.7	59.5	0.25^‡^	
CI	76.7	78.6	1.00^‡^	
Ear to ear distance	32.2	32.7	0.33^**†**^	
Ear to ear index	15.2	15.8	0.56^‡^	
Pupillary distance	5.5	5.9	0.15^**†**^	
Glabella-opisthocranion distance	35.1	35.9	0.72^**†**^	
Forehead to back index	19.7	20.1	0.77^‡^	

* Results of patients with available data for the anthropometric measurements. Total number of treated patients was 7 and total number of non-treated patients was 6. ^**†**^ p-value for independent samples T-test for difference in means. ^‡^ p-value for Mann-Whitney U test for difference in medians. Head circumference, ear to ear distance, ear to ear index, pupillary distance, glabella-opisthocranion distance and forehead to back index are measured in centimeters. CI = cephalic index

Of all study participants (both treated and non-treated), 36 (69.2%) actively participated and filled out and returned the CSO-Q, and 23 (44.2%) attended the follow-up appointment. Of the treated participants, 29 (70.7%) filled out and returned the CSO-Q and 16 (39.0%) attended the follow-up appointment. For these treated patients, the mean follow-up period between surgical treatment and the CSO-Q was 20.2 ± 2.5 years (range 15.9 to 25.9 years) and the mean follow-up period between surgical treatment and the follow-up appointment was 20.9 ± 2.7 years (range 16.4 to 25.9 years).

Of the non-treated participants, 7 (63.6%) filled out and returned the CSO-Q and also attended the follow-up appointment. For these patients, the mean period between diagnosis and CSO-Q was 19.3 ± 5.0 years (range 15.0 to 28.3 years) and 20.1 ± 5.2 years (range 15.0 to 28.7 years) for the follow-up appointment.

#### Anthropometric measurements

In patients diagnosed with sagittal craniosynostosis, the mean CI at follow up was significantly larger in treated patients than in non-treated patients (74.6 versus 69.1, p = 0.04). The mean pupillary distance and forehead to back index were significantly smaller in treated group than in non-treated group (pupillary distance 6.0 cm versus 6.7 cm [p = 0.04] and forehead to back index 19.6 cm versus 21.1 cm [p = 0.03]). We found no statistically significant differences in head circumference, ear to ear distance, ear to ear index, and glabella-opisthocranion distance between treated and non-treated patients (Table 2 A). In the group diagnosed with metopic craniosynostosis, there were no statistically significant differences in anthropometric measurements between treated and non-treated patients (Table 2B).

### Outcomes of the craniofacial surgery outcomes questionnaire

Of the 36 participants who filled out and returned the CSO-Q (both treated and non-treated), 27 (75.0%) were male. Thirty study participants (83.3%) reported a good general health status, 21 (58.3%) graduated high school, 11 (30.6%) had obtained an under- or postgraduate degree, and 28 (77.8%) were employed at the time of returning the CSO-Q (Table 3). Results of part A are presented in Fig. 2A/B.

**Table 3:** General health and socioeconomic status of 36 patients who returned the Craniofacial Surgery Outcomes Questionnaire (CSO-Q) (all data given as number of patients (%) unless otherwise indicated)

Characteristic	Value
General health status*	
Good	30 (83.3)
Fair	4 (11.1)
Bad	1 (2.8)
Social status	
Single	28 (77.8)
Relationship	5 (13.9)
Living together	3 (8.3)
Being a parent	2 (5.6)
Educational attainment*	
Under – or postgraduate degree	11 (30.6)
High school degree	21 (58.3)
In high school	3 (8.3)
Employment status*	
Employed	28 (77.8)
Unemployed	7 (19.4)

*Missing values were present for general health status (2.8%), educational attainment (2.8%), and employment status (2.8%)

Overall, cosmetic satisfaction was seen in 29 participants (80.6%) and cosmetic dissatisfaction in 7 (19.4%). From the commentary sections of the questionnaire, the disappointment described was mainly due to the asymmetry in the face and skull area, with one patient stating a ‘divot’ in the forehead, one patient having a ‘bumpy’ feeling frontally and one patient declaring a ‘pointy forehead’ appearance. Two patients mentioned the ‘visibility’ and ‘wide’ aspect of the scar. Figure 3 shows the results of the RSES. Based on these results, 23 patients (63.9%) were categorized as having high self-esteem, 12 patients (33.3%) were classified as having normal self-esteem, and 1 patient (2.8%) had low self-esteem. The mean RSES score was 25.9 ± 5.3. Patients’ answers to part C are shown in Fig. 4. Based on the results of the FNE scale, 25 patients (69.4%) were classified as having a low FNE, 9 patients (25.0%) showed to have an average FNE, and 2 patients (5.6%) were categorized as having a high FNE. Two patients described in the commentary section that ‘strangers’ often made remarks regarding the asymmetry of their facial appearance and about the scar on the head. The mean score of the FNE scale was 9.3 ± 7.4. We did not find any statistically significant correlations between the total score of part A (cosmetic satisfaction) of the CSO-Q and any of the anthropometric measurements.

Of the 36 study participants, 29 (80.6%) received surgical treatment for their sagittal or metopic craniosynostosis and 7 (19.4%) did not. In these treated versus non-treated study participants, the proportion of cosmetic satisfaction was 24/29 (82.8%) versus 5/7 (71.4%) (p = 0.497). Based on the RSES, 17 treated (58.6%) and 6 non-treated former patients (85.7%) had high self-esteem (p = 0.180), 11 treated (37.9%) and 1 non-treated participant (14.3%) had normal self-esteem (p = 0.384), and 1 treated study participant (3.4%) and none of the untreated participants had low self-esteem (p = 1.000). The mean RSES score for the treated participants was 25.3 ± 5.6 and for the non-treated 28.1 ± 3.1 (p = 0.208). Based on the results of the FNE scale, 19 treated (65.5%) and 6 non-treated study participants (85.7%) had low FNE (p = 0.298); 8 treated study participants (27.6%) and 1 non-treated study participant (14.3%) had average FNE (p = 0.652), and 2 treated study participants (6.9%) and none of the untreated study participants had high FNE (p = 1.000). The mean FNE score for the treated group was 10.0 ± 7.7 and for the non-treated 6.6 ± 5.2 (p = 0.275).

## Discussion

This is, to our best knowledge, the first prospective study of sagittal and metopic craniosynostosis patients regarding long-term anthropometric outcome and patient reported outcome measures, two decades after diagnosis. The study includes patients who were treated surgically and those who received observation only from a single center (BCCH) in the period 1986–2006. Passive participation rate of the study was just over 20% of all former patients. Active participation was a bit lower (~ 15%) which is, in our opinion, a reflection of this kind of studies.

In our study group, significant anthropometric differences, such as the CI and pupillary distance, were found in sagittal craniosynostosis between patients who were operated on versus patients who were left untreated. At the time of our craniosynostosis repair surgeries, techniques were more open repair surgeries as opposed to the more minimal invasive techniques often used nowadays. Nevertheless, we do think these measurements show what could be expected, and still can be expected, when studying long-term outcomes of more current, minimal invasive surgical techniques.

In our cohort of patients diagnosed with metopic craniosynostosis, there were no statistically significant differences found in the long-term anthropometric measurements, between treated and non-treated patients. At infancy, severity of cosmetic dissatisfaction, was highly subjective (if at all expressed by parents) and no categorizable data could be retrieved from the charts. Our anthropometric end outcomes in metopic craniosynostosis might be a reflection of the possibility that patients who were considered ‘severe’ were operated on, and patients who were considered ‘mild’ were left for observation.

Focusing more on the PROMs of our study participants, overall cosmetic satisfaction was found to be high (80.6%) and no differences were found between sagittal and metopic synostosis patients, nor between treated or non-treated craniosynostosis patients. Overall outcomes regarding self-esteem and FNE were comparable to population-based outcomes. Mean RSES scores from an American study in 2010 were 22.62 ± 5.80 [9] and mean FNE found in a French study was 12.1 ± 6.1. [10] In our cohort we found at least equivalent or even slightly better means of RSES and FNE, i.e. 25.9 ± 5.3 and 9.3 ± 7.4, respectively. Very importantly, the data suggest that despite somewhat poorer anthropometric results in the untreated sagittal synostosis patients, overall cosmetic satisfaction, self-esteem and fear of negative evaluation at adolescent/adult age did not differ from the treated group of patients.

Limitations of this study exist. The study cohort size is moderate and due to the study’s long-term nature, it was expected that only a subset of former patients could be contacted. Since there were no incentives to participate, as expected, a modest number of patients were willing to share the patient data already available in their clinical records and fewer participated actively in the study at adult age, often more than two decades after their sagittal or metopic craniosynostosis diagnosis and treatment. This study assessed the patient’s own perception of their long term outcomes. While there may have been biases on the part of the decision makers in infancy (the treating physician and the parents), and this may have influenced the patient’s own perceptions during childhood and adolescence, it is likely of little relevance to these results.

The outcome of this study might have been influenced by lower study participation rates in the not surgically treated group of former patients. Although we do not know why the untreated patients had a lower participation rate, one might speculate that these patients did not have as strong a bond with the surgeons as those who had received surgery and they just did not wish to be bothered. Perhaps, they had chosen to ignore and forget their diagnosis and they did not wish to create unnecessary anxiety for themselves by participating in a study.

Nevertheless, we think this study still provides valuable long-term data on patient reported outcomes in sagittal and metopic craniosynostosis treated with repair surgeries or observation only. Furthermore, our data provide a platform from which one can develop an inclusive and uniform approach to assess patient’s subjective cosmetic satisfaction using the CSO-Questionnaire. This study might also help to optimize preoperative counseling and psychosocial care for patients and their families.

## Conclusion

This is the first prospective study of sagittal and metopic craniosynostosis patients regarding long-term anthropometric outcome and patient reported outcomes measures, including patients who were treated surgically and those who received observation only. Although study participation two decades after initial diagnosis was difficult to obtain, our data provide a platform from which one can develop an inclusive and uniform approach to assess patients’ subjective cosmetic satisfaction using the CSO-Questionnaire and might be helpful in preoperative counseling and psychosocial care for patients and their families.

**Fig. 1A:**
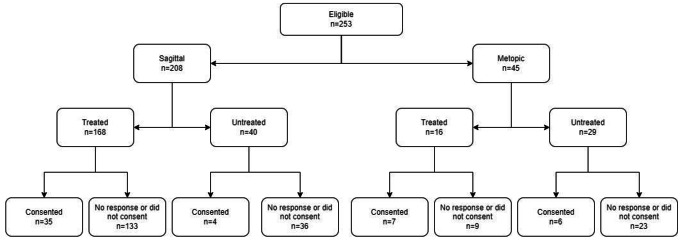
Flow-diagram of patients that consented for the study

**Fig. 1B:**
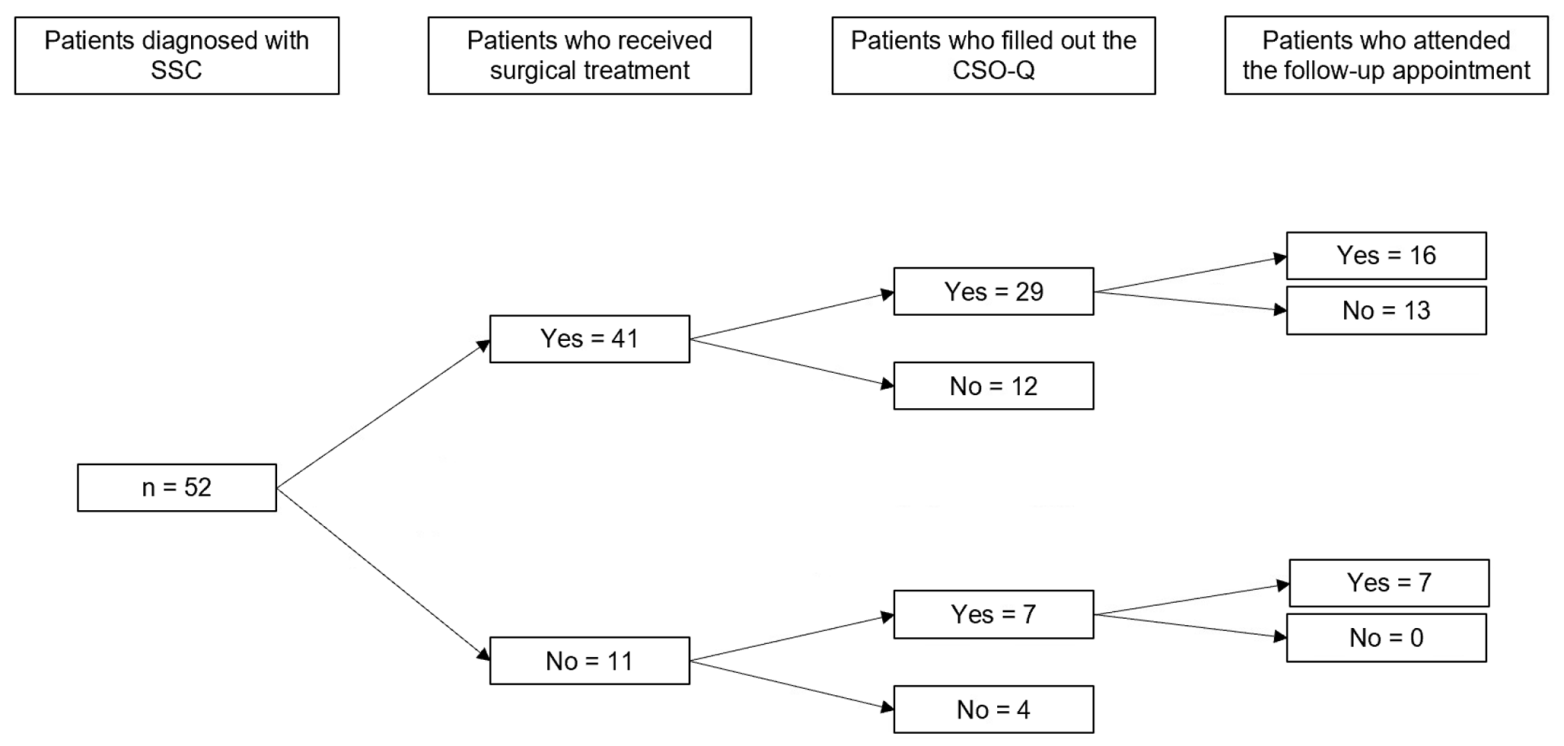
Flow-diagram of study participants

**Fig. 2A:**
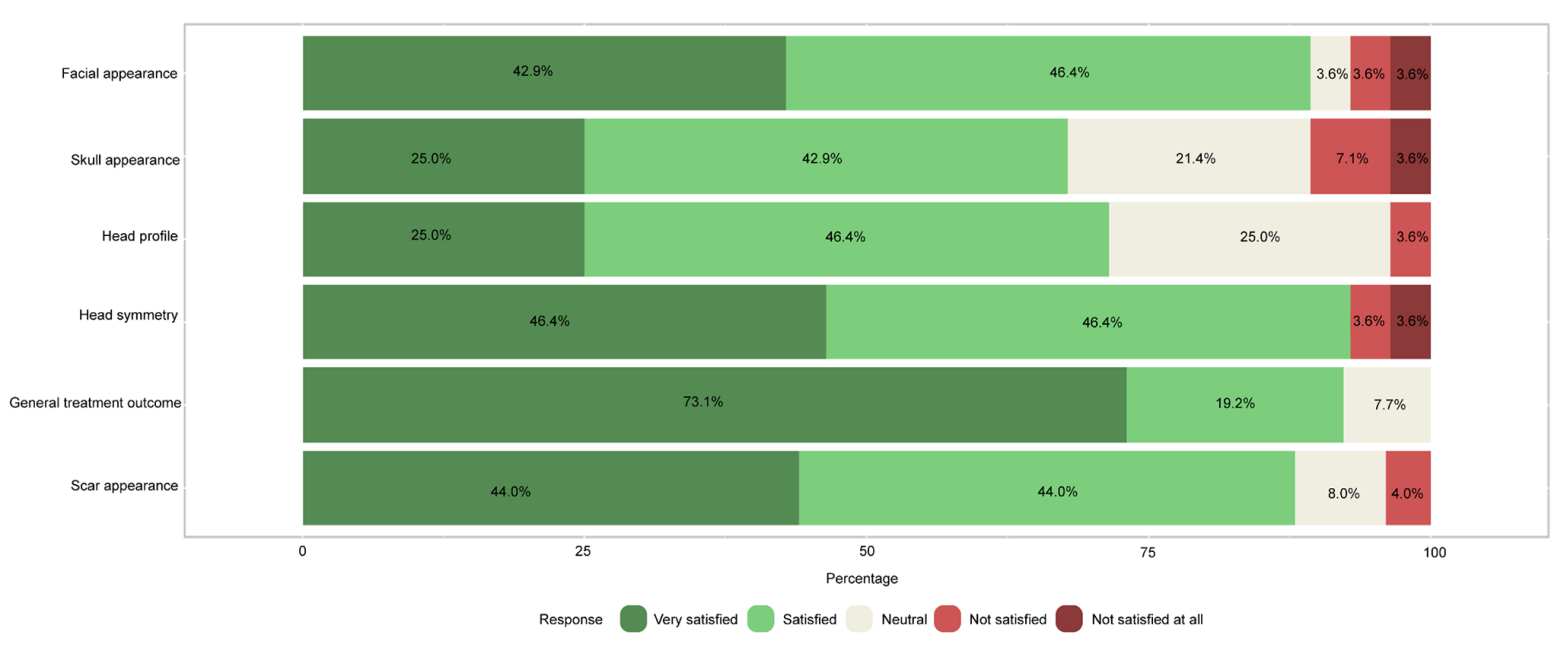
Likert scale of satisfaction with the aesthetic appearance of the face and skull (Part A) of 29 study participants (sagittal craniosynostosis) who returned the Craniofacial Surgery Outcomes Questionnaire (CSO-Q).

**Fig. 2B:**
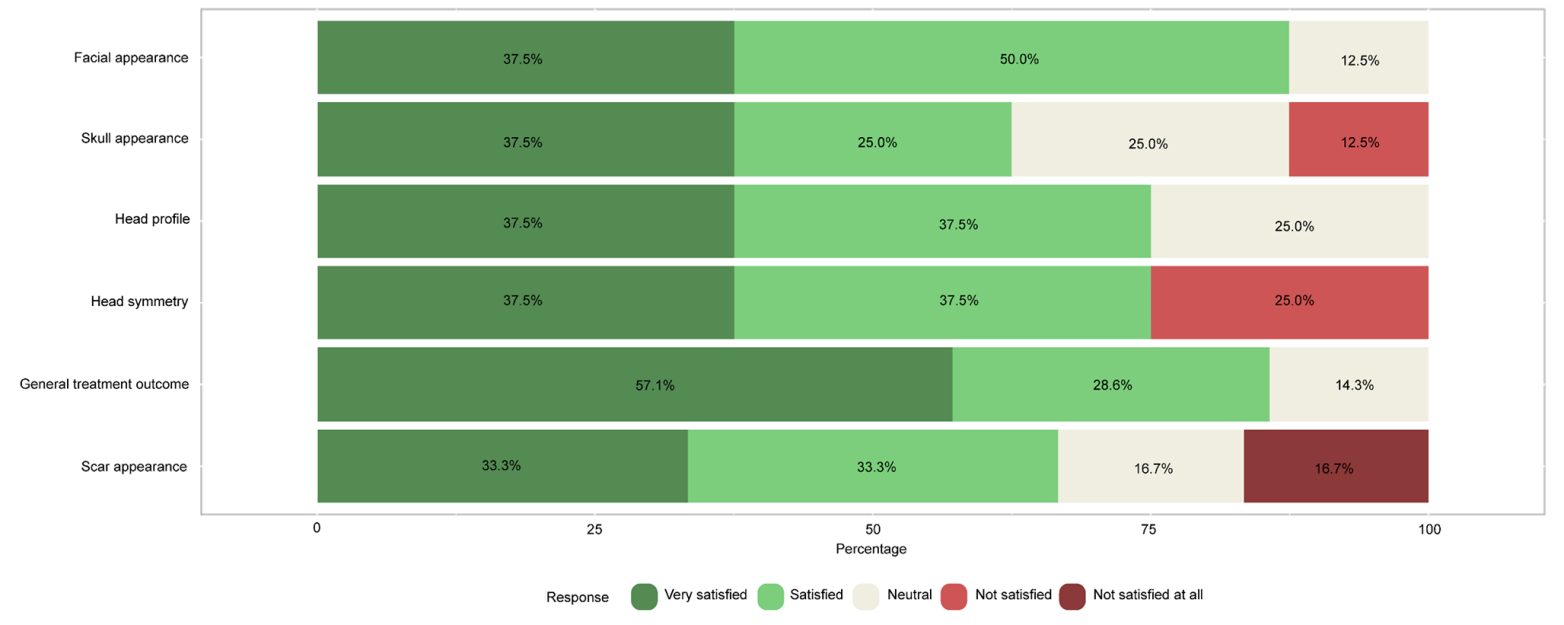
Likert scale of satisfaction with the aesthetic appearance of the face and skull (Part A) of 7 study participants (metopic craniosynostosis) who returned the Craniofacial Surgery Outcomes Questionnaire (CSO-Q).

**Fig. 3:**
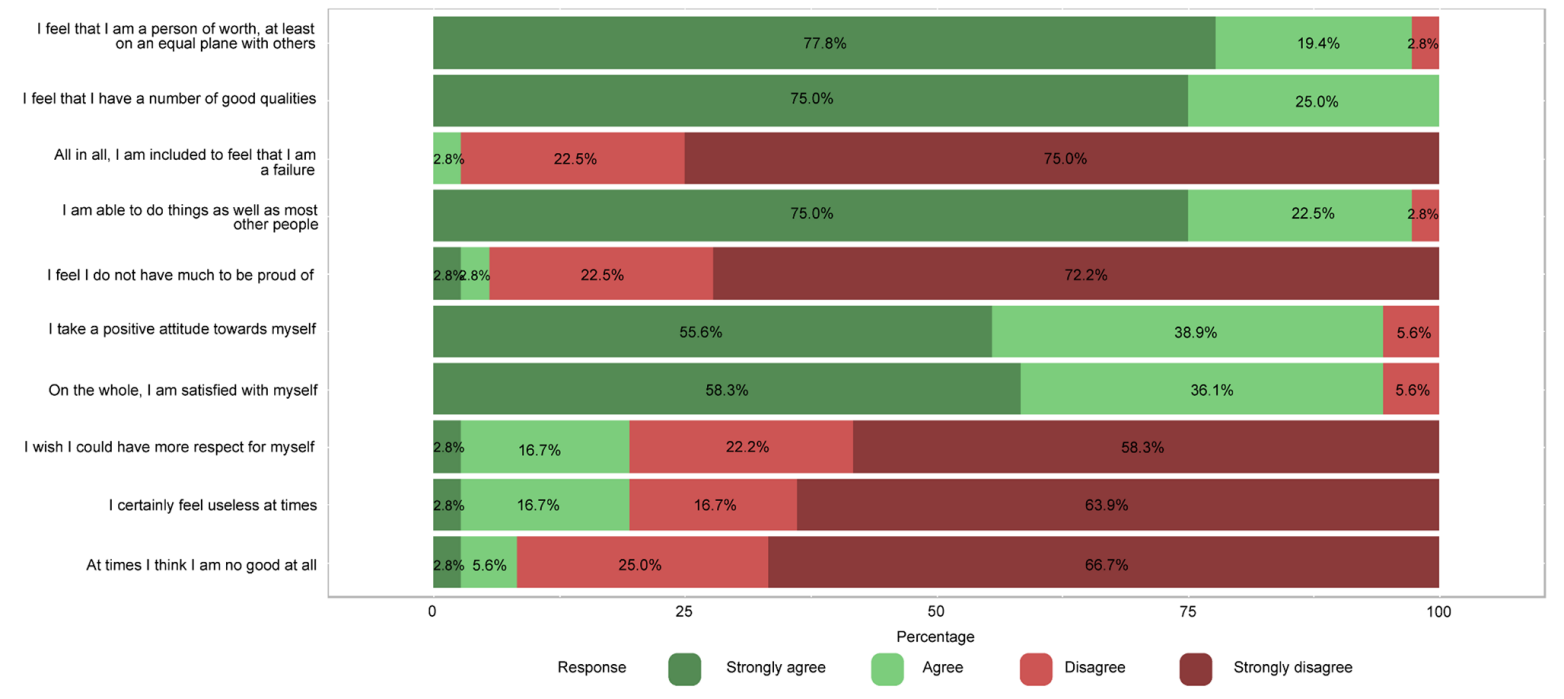
Likert scale of the Rosenberg Self-Esteem Scale (RSES) of 36 patients who returned the Craniofacial Surgery Outcomes Questionnaire (CSO-Q).

**Fig. 4:**
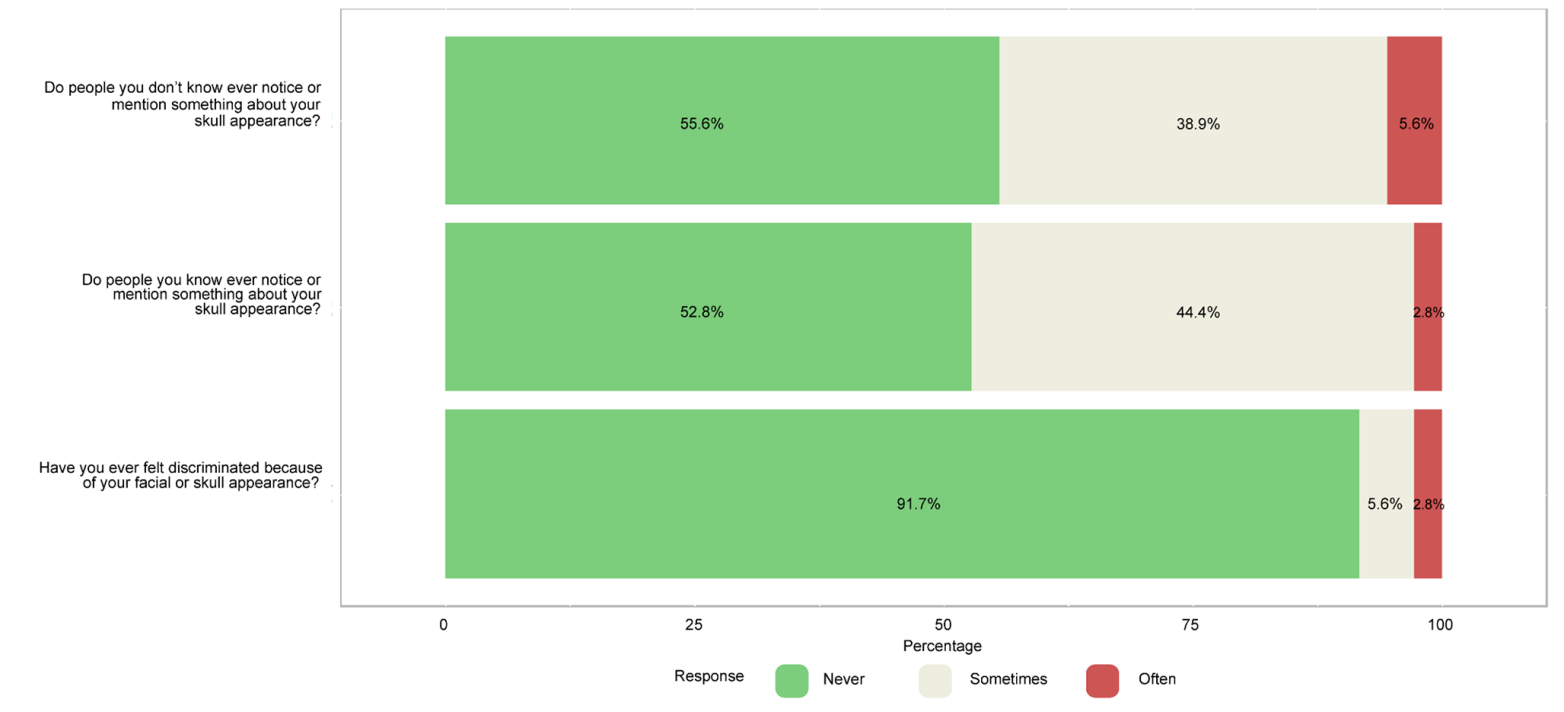
Likert scale of patients’ feelings of noticeability of facial and skull appearance to others of 36 patients who returned the Craniofacial Surgery Outcomes Questionnaire (CSO-Q).

### Electronic supplementary material

Below is the link to the electronic supplementary material.

Supplementary Material 1

## Data Availability

The datasets used and/or analyzed during the current study are available from the corresponding author on reasonable request.

## Abbreviations

PROM: patient-reported outcome measure
SSC: single-suture craniosynostosis
BCCH: British Columbia Children’s Hospital
CSO-Q: Craniofacial Surgery Outcomes Questionnaire
RSES: Rosenberg Self-Esteem Scale
FNE: fear of negative evaluation
MREC: Medical Research Ethics Committee
SCS: sagittal craniosynostosis
SD: standard deviation
IQR: interquartile ranges
CI: cephalic index
